# Optimization of Natural Lipstick Formulation Based on Pitaya (*Hylocereus polyrhizus*) Seed Oil Using D-Optimal Mixture Experimental Design

**DOI:** 10.3390/molecules191016672

**Published:** 2014-10-16

**Authors:** Norsuhaili Kamairudin, Siti Salwa Abd Gani, Hamid Reza Fard Masoumi, Puziah Hashim

**Affiliations:** 1 Halal Products Research Institute, Universiti Putra Malaysia, Putra Infoport, 43400 UPM Serdang, Selangor, Malaysia; E-Mail: puziah.hashim@gmail.com; 2 Centre of Foundation Studies for Agriculture Sciences, Universiti Putra Malaysia, 43400 UPM Serdang, Selangor, Malaysia; 3 Department of Chemistry, Faculty of Sciences, Universiti Putra Malaysia, 43400 UPM Serdang, Selangor, Malaysia; E-Mail: fardmasoumi@upm.edu.my

**Keywords:** cosmetics, lipstick formulation, pitaya seed oil, D-optimal, mixture design

## Abstract

The D-optimal mixture experimental design was employed to optimize the melting point of natural lipstick based on pitaya (*Hylocereus polyrhizus*) seed oil. The influence of the main lipstick components—pitaya seed oil (10%–25% w/w), virgin coconut oil (25%–45% w/w), beeswax (5%–25% w/w), candelilla wax (1%–5% w/w) and carnauba wax (1%–5% w/w)—were investigated with respect to the melting point properties of the lipstick formulation. The D-optimal mixture experimental design was applied to optimize the properties of lipstick by focusing on the melting point with respect to the above influencing components. The D-optimal mixture design analysis showed that the variation in the response (melting point) could be depicted as a quadratic function of the main components of the lipstick. The best combination of each significant factor determined by the D-optimal mixture design was established to be pitaya seed oil (25% w/w), virgin coconut oil (37% w/w), beeswax (17% w/w), candelilla wax (2% w/w) and carnauba wax (2% w/w). With respect to these factors, the 46.0 °C melting point property was observed experimentally, similar to the theoretical prediction of 46.5 °C. Carnauba wax is the most influential factor on this response (melting point) with its function being with respect to heat endurance. The quadratic polynomial model sufficiently fit the experimental data.

## 1. Introduction

Cosmetics are substances that are used to improve female appearance and involve one of the most successful industries in the world. Every day, many new cosmetics products are produced and are improved in comparison to previous ones. Examples of cosmetics are lotions, powders, lipsticks and others. Deodorants, baby products, bath oil, bubble bath products, bath salts, butters and other types of products are in great demand in both developing and developed countries [1]. Cosmetics products are directly applied on the outer surface of human skin. Human skin acts as protective barrier, through which certain ingredients may penetrate [2]. Therefore, consumers are searching for natural-based cosmetics to avoid allergic reactions, any side effects and for the safety of their health. The important criteria for the cosmetics formulation are the raw materials, which can be either synthetics or natural materials.

Lipstick is a lip coloring that has its earliest use dating back to the prehistoric age. In the present day, the use of this product has increased, and the choices of shades of colors, textures and luster have changed and widened. This can be seen from the fact that lipstick is now marketed with hundreds of shades of colors to satisfy the demands of women [3]. A good lipstick should have persuading characteristics and be multifunctional in order to be acceptable to consumers, such as having a suitable texture and antioxidant properties. Emulsifiers, emollients, binders and colorants are among the variety of components that contribute to the properties of fine lipstick. Texture, melting point and hardness of the lipstick are the influential characteristics that are altered by varying the ratio of the ingredients that are used in the formulation.

Recently, there has been research about many vegetable oils for which each of the oils has its own beneficial effect on the human body. In this present work, we introduce *Hylocereus polyrhizus* oil as a new oil in our natural lipstick formulation. *Hylocereus polyrhizus* belongs to the Cactaceae family. It has gained popularity in many countries in Asia. Commercial cultivation of pitaya is expanding in several parts of Asia, such as Malaysia, Vietnam and the Philippines [4]. Due to its bright red skin with green, overlapping pins covering the fruit, Asian people have called it “dragon fruit”. Pitaya comes in a number of varieties; each may be distinguished from the other by either the color of the pulpy skin (exocarp) and/or the color of the soft fleshy center (mesocarp or endocarp), which contains the seed [5]. Many health benefits have been reported regarding red pitaya, such as cancer chemoprevention, anti-inflammatory, anti-diabetic and cardiovascular mortality risk reducing properties [6]. In this work, we use the pitaya seed oil in our formulation, because it contains a significantly high content of linoleic acid and linolenic acid, which are unsaturated fatty acids (UFAs) [5]. These UFAs help to balance the skin’s metabolism by controlling the flow of oils and nourishing collagen, the supporting structure beneath the skin. Incorporating the sought-after essential oil, omega-3, in food or cosmetic products is widely practiced [7].

A common issue in the pre-formulation of cosmetic products, including lipstick, is the optimization of the mixture composition, which aims to obtain a product with the required characteristics. A statistical design is a solution to overcome this issue. Several statistical experimental design techniques were used for the formulation work, such as a factorial design, a cross design and a mixed experimental design [8]. The D-optimal mixture experimental design encompasses one of the most effective tools for solving such optimization issues and the functional stability of the influencing factors. The early goal of using this tool was to minimize the effort, time and resources involved while obtaining a valid result [9]. Therefore, the D-optimal mixture design method is an adequate and more effective method compared to previous methods, such as the classical one-variable-at-a-time method, because it can study many variables simultaneously with a low number of observations, as well as saving time and costs [8]. In previous works, this tool has proven to be an effective tool to study the relationship between variables in formulation work [10]. However, cosmetic chemists rarely have used this tool.

Therefore, the aim of this work is to use the D-optimal mixture design as a tool to optimize the natural lipstick evaluation and to evaluate simultaneously the interaction effects between the factors, including several oils, waxes and other materials, as well as to study which variables influence the melting point.

## 2. Results and Discussion

### 2.1. Screening Variables

The preliminary study was carried out in order to determine the levels of independent variables. Based on the resultant data, the lower and upper limits of the five independent components were determined. The lipstick formulation demonstrated a melting point above 40 °C by restraining the range of pitaya seed oil, virgin coconut oil, beeswax, candelilla wax and carnauba wax to levels of 10%–35%, 25%–45%, 5%–25%, 1%–5% and 1%–5%, respectively.

### 2.2. Fitting the Models

Variation in the melting point was predicted by employing the D-optimal mixture design as the response function of the lipstick pitaya seed oil-based composition in the formulation. The effects of five independent variables, namely pitaya seed oil (A), virgin coconut oil (B), beeswax (C), candelilla wax (D) and carnauba wax (E), on the melting point (Y) were investigated using the D-optimal mixture design, as shown in Table 1.

Thus, the experimental data were analyzed statistically. Statistics was used to find the best fitted model for five independent variables. The significance of the coefficient of the quadratic polynomial models was evaluated by using ANOVA [11]. For any terms in the models, a large *F*-value and a small *p*-value indicated a more significant effect on the respective response variables.

**Table 1:** Experimental data and response (melting point) obtained from the mixture design. Letter code: pitaya seed oil (**A**), virgin coconut oil (**B**), beeswax (**C**), candelilla wax (**D**) carnauba wax (**E**), melting point (**Y**).

Experiment No.	A	B	C	D	E	Melting Point (Y): (°C)
1	28.727	25.203	24.993	3.077	1.000	47.0
2	35.000	33.212	8.494	4.997	1.297	41.0
4	10.079	40.854	25.000	2.647	4.42	50.0
5	35.000	33.212	8.494	4.997	1.297	41.0
6	10.185	39.743	25.000	4.985	3.087	49.0
7	26.412	25.600	25.000	1.001	4.986	49.0
8	22.989	44.847	5.166	5.000	4.998	44.0
9	10.071	45.000	17.959	4.984	4.986	50.5
10	24.427	29.360	21.110	5.000	3.102	50.0
12	14.997	45.000	16.945	1.065	4.993	48.0
13	10.908	44.982	25.000	1.11	1.000	51.0
14	26.412	25.600	25.000	1.001	4.986	49.0
16	34.987	33.146	8.289	1.578	5.000	43.0
17	29.985	38.673	12.041	1.301	1.000	45.0
19	17.756	44.997	15.384	3.863	1.000	46.0
20	19.069	37.65	19.839	1.977	4.465	51.0
21	28.727	25.203	24.993	3.077	1.000	45.0
22	26.866	34.355	18.681	1.000	2.097	45.0
24	34.987	33.146	8.289	1.578	5.000	41.0
25	10.908	44.982	25.000	1.110	1.000	48.0

Table 2 shows the analysis of variance (ANOVA) for the quadratic model. The *R*^2^, adjusted *R*^2^, predicted *R*^2^, regression (*p*-value) and *F*-value are also shown in Table 2. It should be mentioned that non-significant (*p* > 0.05) linear terms were included in the final reduced model if the quadratic or interaction terms containing these variables were found to be significant (*p* < 0.05) [12]. In this work, the D-optimal analysis demonstrated that the second-order polynomial used for melting point determination is *R^2^* = 0.9267. The resultant coefficient showed that more than 90% of the response variation of the melting point could be described by D-optimal mixture design models as the function of the main lipstick formulation. It was observed that a lack of fit had no indication of significance (*p* > 0.05) for the final model. Therefore, the satisfactory fitness of the model to the significant (*p* < 0.05) factors’ effects (Table 2) was proven. As stated in Table 2, the model *F*-value of 14.04 indicates that the model is significant. There is only a 0.01% chance that a “model *F*-value” this large could happen due to noise. When the value of “probability > *F*” was less than 0.05, this implies that the model terms are significant. In this case, the interactions between AC, BD and BE are significant (*p* < 0.05) model terms. The model terms are considered not significant when the values are greater than 0.1000.

From Table 3, we observed that the linear coefficient and quadratic coefficient were obtained. The final model to predict the melting point of the natural lipstick formulation is shown in regression Equation (1). A positive value in the regression equation represents an effect that favors optimization due to a synergistic effect, while a negative value indicates an inverse relationship or antagonistic effect between the factor and response [13]. It was observed that all of the linear coefficients of the model and one quadratic coefficient (A and C) gave a positive effect. The biggest effect on the melting point is from E (carnauba wax) due to its function in heat endurance [14]. The negative values of the coefficient estimation denote a negative influence of the variables on the formulation. In this case, the interaction between A and E, B and D, and C and E gave negative effects on the formulation.

Y = 51.55A + 62.05B + 34.31C + 46.28D + 278.76E + 15.21AC − 314.07AE − 102.29BD − 369.95BE − 263.64CE
(1)

**Table 2:** Analysis of variance (ANOVA) for the D-optimal mixture design of the quadratic model.

Source	Sum of Square	df	Mean Square	*F*-Value	Probability > F	Significance
Model	203.99	9	22.67	14.04	0.0001	Significant
Linear Mixture	184.57	4	46.14	28.58	<0.0001	
AC	8.06	1	8.06	4.99	0.0495	
AE	6.51	1	6.51	4.03	0.0724	
BD	10.62	1	10.62	6.58	0.0281	
BE	8.21	1	8.21	5.09	0.0477	
CE	5.5	1	5.5	3.41	0.0946	
Residual	16.15	10	1.61			
Lack of Fit	7.65	5	1.53	0.9	0.5449	Not significant
Pure Error	8.5	5	1.7			
Cor Total	220.14	19				
*R*^2^	0.9267					
*R*^2^ (Predicted)	0.7306					
*R*^2^ (Adjusted)	0.8606					
Regression (*p*-value)	0.0001					
Lack of Fit (*p*-value)	0.5449					

**Table 3:** Regression coefficient values for the final reduced model.

Source	Coefficient Estimate
A	51.55
B	62.05
C	34.31
D	46.28
E	278.76
AC	15.21
AE	−314.07
BD	−102.29
BE	−369.95
CE	−263.64

### 2.3. D-Optimal Analysis

In general, there is high demand in the cosmetic industry for natural-based lipstick with good characteristics. A high melting point of lipstick is good for avoiding technical deterioration when exposed to the anticipated environmental temperature and humidity during preparation and use.

**Figure 1:**
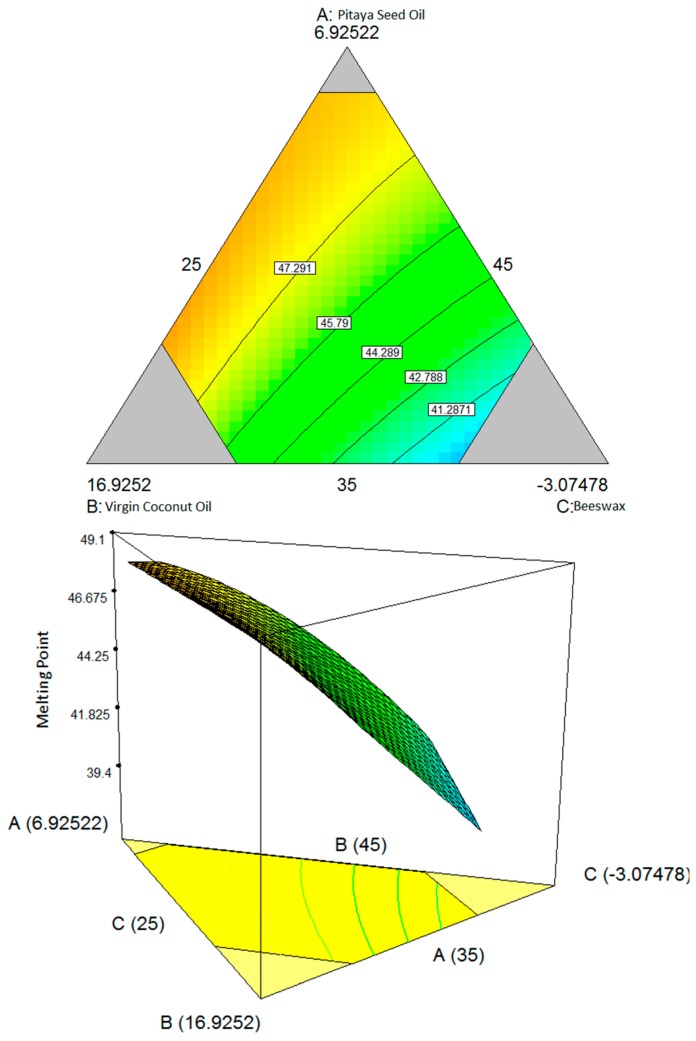
Contour plot (two-dimensional) and three-dimensional surface plots showing the interaction effect between three variables: (**A**) pitaya seed oil, (**B**) virgin coconut oil, (**C**) beeswax; and two variables are kept constant: (**D**) candelilla wax and (**E**) carnauba wax, with respect to the melting point.

As shown in Figure 1, the amount of both oils (pitaya seed oil and virgin coconut oil) produced a minimum effect on the melting point of the lipstick. However, an increasing in the beeswax percentage also increased the melting point of the lipstick. This means that beeswax improves the melting point of the lipstick.

X1 = A: Pitaya seed oil; X2 = B: Virgin coconut oil; X3 = E: Beeswax; Actual components: B: Candelilla wax = 3.037; C: Carnauba wax = 3.037.

**Figure 2:**
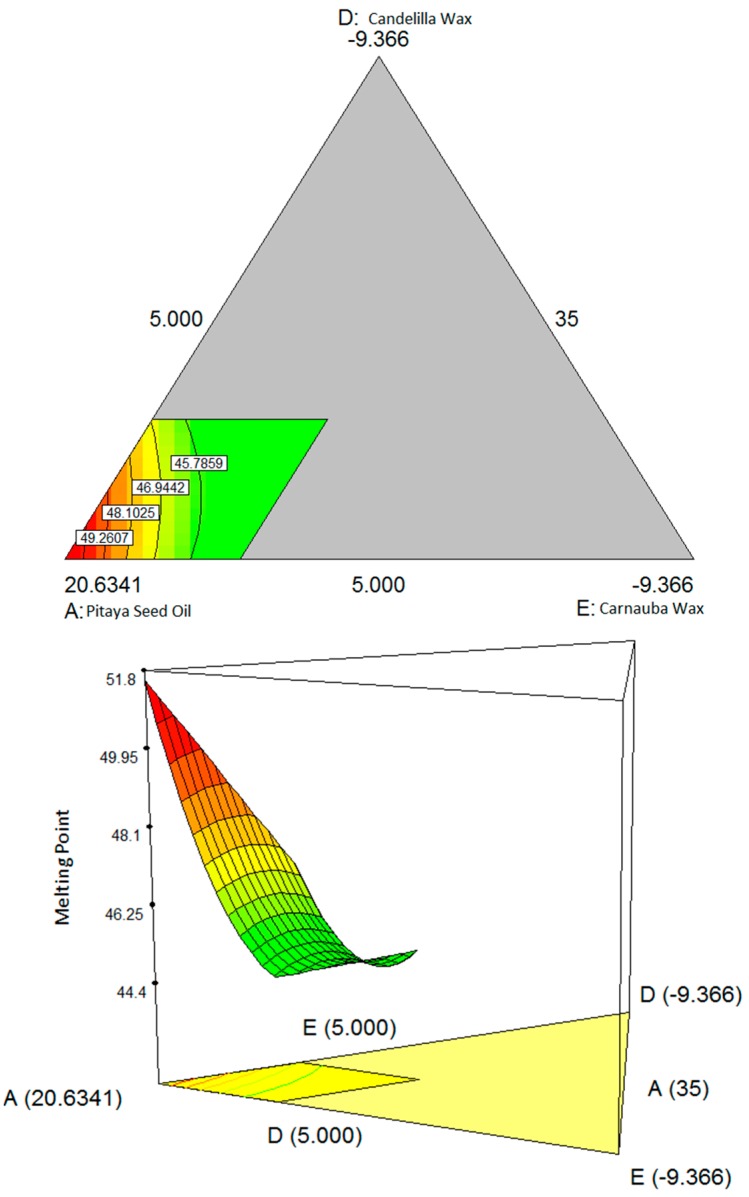
Contour plot (two-dimensional) and three-dimensional surface showing the interaction effect between three variables: (**A**) pitaya seed oil, (**D**) candelilla wax, (**E**) carnauba wax; and (**B**) virgin coconut oil and (**C**) beeswax are kept constant respect to melting point.

Figure 2 shows the interaction between the pitaya seed oil, carnauba wax and candelilla wax. The higher amount of carnauba wax and candelilla wax gives a higher value for the melting point [8]. This is due to the function of these waxes, which is to confer a high melting point to the finished product [15].

X1 = D: Candelilla wax; X2 = A: Pitaya seed oil; X3 = E: Carnauba wax; Actual components: B: Virgin coconut oil = 36.183; C: Beeswax = 16.183.

### 2.4. Optimization of D-Optimal Mixture Design for Formulating Lipstick

Using Design-Expert software, the desirability function was investigated to obtain an optimized formulation. An optimum lipstick formulation is that with a melting point from 40–56 °C, as stated by previous studies and commercialized products. D-optimal surface and contour plots were used to visualize the interaction between the independent variables. By investigating the interaction effect between independent variables and evaluating the optimization constraints, the optimum lipstick formulation was prepared with 37.0% virgin coconut oil, 25.0% pitaya seed oil, 17.0% beeswax, 2.0% candelilla wax and 2.0% carnauba wax. Based on the optimum formulation, the predicted value of the melting point is 45.5 °C.

### 2.5. Verification of Reduced Model

The experimental and predicted values of the response were compared to check the adequacy of the D-optimal surface equation. The optimized lipstick formulation has a melting point of 46.0 °C. As displayed in Table 4, no significant difference was noted between the theoretical predicted and experimental value under optimal conditions. The experiments were formulated under the recommended conditions, and the resulting responses were compared to the predicted ones by computing the residual standard error, as shown below:

Residual Standard Error (RSE) % = Experimental Value – Predicted Value ÷ Predicted Value × 100 [16].

**Table 4:** Predicted and observed values for optimal formulation.

Independent Variables					Melting Point (°C)		RSE (%)
A (%)	B (%)	C (%)	D (%)	E (%)	Predicted	Experimental	
25	37	17	2	2	45.5	46	1.099

## 3. Experimental Section

### 3.1. Materials

Pitaya seed was purchased from a local company in Malaysia, Great Sun Pitaya Farm, Teluk Panglima Garang, Selangor, Malaysia. The compositions of pitaya seed oil are saturated fatty acid (23.6%), monounsaturated fatty acid (25.6%) and polyunsaturated fatty acid (50.8%). N-Hexane and ethanol purchased from Merck Chemicals, Darmstadt, Germany. Virgin coconut oil and castor oil were purchased from Euro-Pharma Sdn Bhd, Pulau Pinang, Malaysia. Beeswax, candelilla wax and carnauba wax were purchased from Making Cosmetics Inc., Snoqualmie, WA, USA. Red iron (III) oxide was purchased from Sigma-Aldrich, St. Louis, MO, USA. All others chemicals and ingredients used were of analytical, food or cosmetics grade.

### 3.2. Extraction of Pitaya Seed Oil

Solvent extraction was carried out to extract the compounds from pitaya seed. This extraction technique used *n*-hexane and ethanol as the solvents to extract the unsaturated fatty acids and antioxidants (flavonoid and phenol) from pitaya seed. The extraction process was as follows; pitaya seed (20 g) was ground using a blender, soaked in the *n*-hexane and ethanol (450 mL) and left overnight. Upon completion of the oil extraction, the *n*-hexane and ethanol were discarded from the oil in a rotary evaporator. The process was repeated three times in order to make sure all of the unsaturated fatty acids were extracted from the seed.

### 3.3. Formation of Lipstick

Lipstick was formulated using a mixture of natural ingredients, which were a blend of several oils, natural waxes and others materials with protective properties prepared at the laboratory scale. Firstly, the colorant powder was added into the homogenized oil phase in order to ensure the dispersion of the pigment [15]. The solution (A) was homogenized using a high speed homogenizer (IKA^®^ T18 Basic ULTRA-TURRAX, Hamburg, Germany) at a speed of 10,000 rpm. The waxes (B) were added into the solution (A) and heated to 85–90 °C until the waxes melted. The mixtures (A and B) were homogenized together, and the protective materials are added at the end. All of the ingredients were homogenized at a speed of 10,000 rpm for 40 min at 70–80 °C. Then, the hot mixture was poured into the lipstick mold and kept at −2 °C for 2 h.

### 3.4. Preliminary Study

The preliminary formulation of the lipstick was prepared in order to obtain a suitable range to be added in the mixture design. The optimum formulation that was suggested in previous work consists of 39.40% castor oil, 20.00% beeswax, 5.0% carnauba wax, 5.00% candelilla wax and 17.6% solvent [8]. Thus, six preliminary formulations were made according to this previous work in order to obtain the lowest limit and highest limit for the amounts to be added in the D-optimal design.

### 3.5. Experimental Design

The experimental mixture design was utilized to study the effect of a five-component system—pitaya seed oil (A), virgin coconut oil (B), beeswax (C), candelilla wax (D) and carnauba wax (E)—on the response variable: the melting point (Y). Hence, based on the design of the software, a total of 25 experiments were run using Design Expert software (version 7, Stat-Ease Inc., Minneapolis, MN, USA). The D-optimal criterion was used to select a set of candidate points in the design space. In D-optimal design, there are restrictions on the component proportions *Xj* that take the form of lower (*Lj*) and upper (*Uj*) constraints, to keep the experimenter from exploring the entire simplex region. The general form of constraints in D-optimal design is as follows: ∑ *Xj* = 1 and *Lj* ≤ *Xj* ≤ *Uj*

The constraints of the component proportions are shown in Table 5. These lower and upper limits of *Xj* are chosen to describe the behavior of the formulations, which have compositions close to that of the best experiment obtained from preliminary work. In this work, given 25-candidate experiments, a 20-run D-optimal mixture experimental design resulted to be the best subset according to the information matrix determination maximization criterion [17]. Table 6 reports the 20-run experimental plan in which the mixture component proportions (% w/w) are indicated. According to this experimental plan, the test mixtures were prepared and analyzed for melting point.

**Table 5:** Constraints of the independent variable proportion.

Independent Variables, *Xi*	Lower Limit, *Lj*	Upper Limit, *Uj*
Pitaya seed oil, A	10	35
Virgin coconut oil, B	25	45
Beeswax, C	5	25
Candelilla wax, D	1	5
Carnauba wax, E	1	5

### 3.6. Melting Point Evaluation of Lipstick

It is very necessary to maintain a constant standard for lipstick, and keeping this view in mind, the formulated lipstick was evaluated with respect to important parameters, such as the melting point. The melting point is one the most important determinations, due to the indication of the limit on its safe storage. The melting point of the formulated lipstick was determined using the slip melting point method, which is one of the conventional techniques to determine the melting point of a waxy solid. It was determined by casting a 10-mm column of the solid in a capillary tube with an internal diameter of about 1 mm and a length of about 80 mm. Then, it was immersed in a temperature-controlled water bath. The melting point is the temperature at which the product is slowly melted out of the tube. This procedure was repeated 3 times to obtain the average and consistency.

**Table 6:** The D-optimal design with 20 out of 25 experiments.

Experiment No.	Blocks	A	B	C	D	E
1	Block 1	28.727	25.203	24.993	3.077	1.000
2	Block 1	35.000	33.212	8.494	4.997	1.297
4	Block 1	10.079	40.854	25.000	2.647	4.42
5	Block 1	35.000	33.212	8.494	4.997	1.297
6	Block 1	10.185	39.743	25.000	4.985	3.087
7	Block 1	26.412	25.600	25.000	1.001	4.986
8	Block 1	22.989	44.847	5.166	5.000	4.998
9	Block 1	10.071	45.000	17.959	4.984	4.986
10	Block 1	24.427	29.360	21.110	5.000	3.102
12	Block 1	14.997	45.000	16.945	1.065	4.993
13	Block 1	10.908	44.982	25.000	1.11	1.000
14	Block 1	26.412	25.600	25.000	1.001	4.986
16	Block 1	34.987	33.146	8.289	1.578	5.000
17	Block 1	29.985	38.673	12.041	1.301	1.000
19	Block 1	17.756	44.997	15.384	3.863	1.000
20	Block 1	19.069	37.65	19.839	1.977	4.465
21	Block 1	28.727	25.203	24.993	3.077	1.000
22	Block 1	26.866	34.355	18.681	1.000	2.097
24	Block 1	34.987	33.146	8.289	1.578	5.000
25	Block 1	10.908	44.982	25.000	1.110	1.000

### 3.7. Statistical Analysis

In the mixture design, the optimal conditions of the independent variables were found to predict the variation of the material compositions, as well as the preparation conditions. The optimal conditions and preparation of natural lipstick were chosen based on the condition of attaining a medium melting point (*R*_1_). By using the quadratic regression equation, the D-optimal model for five components takes the form of the following equation:

*Y* = *b* + *b*_1_*X*_1_ + *b*_2_*X*_2_ + *b*_3_*X*_3_ + *b*_4_*X*_4_ + *b*_5_*X*_5_ + *b*_12_*X*_1_*X*_2_ + *b*_13_*X*_1_*X*_3_ + *b*_14_*X*_1_*X*_4_ + *b*_15_*X*_1_*X*_5_ + *b*_23_*X*_2_*X*_3_ + *b*_24_*X*_2_*X*_4_ + *b*_25_*X*_2_*X*_5_ + *b*_34_*X*_3_*X*_4_ + *b*_35_*X*_3_*X*_5_ + *b*_45_*X*_4_*X*_5_ (2)

where *y* is the predicted response, *b* is a constant and *b_i_*, *b_ii_* and *b_ij_* are the linear, quadratic and interaction coefficients, respectively [18]. The statistical significance between the independent variables was determined by utilizing analysis of variance (ANOVA). The significant (*p* < 0.05) independent variables were only involved in the reduced model, and non-significant (*p* > 0.05) independent variables were eliminated. It was suggested that *R*^2^ should be at least 0.08, for a good fit of the model [18].

### 3.8. Verification of the Models

A quantitative comparison between the obtained experimental and theoretical prediction values was made to validate the models. The percentage of the calculated value was also determined. The predicted error is the difference between the experimental value and the predicted value per predicted value [19].

## 4. Conclusions

The current study showed that the D-optimal mixture experimental design is an effective and beneficial tool for carrying out the optimization study of natural lipstick formulations by combining the independent variables: virgin coconut oil, pitaya seed oil, beeswax, candelilla wax and carnauba wax. The optimum components for the natural lipstick formulation were established. The effects of the mixture components on the physical properties of the formulation have been investigated using D-optimal design. The analysis of variance stated that the accuracy of the model, using a low *F*-value (14.04) and a very low *p*-value (<0.0001) with a non-significant lack of fit, and *R*^2^ = 0.9267. This research furnished a guideline to improving the specific desirable characteristics by using the D-optimal design, which is allows studying many variables simultaneously. The research findings also provide a guideline of the effects of ingredients on the physical properties.
